# Supplementary material for: Negotiating illness through faith: Religious narratives of cancer etiology among patients and caregivers in Addis Ababa, Ethiopia
Source: PLoS One. 2026 Jun 5;21(6):e0349510. doi: 10.1371/journal.pone.0349510 (PMC13240894; doi:10.1371/journal.pone.0349510)
Supplement: S1 File — (DOCX) [file pone.0349510.s001.docx]

**Note on the Transcription Content**

This supplementary file presents selected English-language transcriptions from in-depth interviews conducted for this study. The excerpts included here focus specifically on participants’ religious narratives regarding illness etiology and religiously informed care-seeking behaviors. Only interviews and segments in which participants explicitly reflected on these themes are presented.

Other portions of the interviews addressing biomedical, social, and personal experiences are not included in this file, as they fall outside the primary analytical focus of the manuscript. The selective inclusion of data was guided by the study’s research objectives and analytical framework and does not indicate the absence of additional relevant material in the full dataset.

**Participant 1 men caring for his mother diagnosed with cancer, Oromia region, Orthodox Christian**

A rural person eats whatever they can find; they don't do anything special. They usually drink milk because it is said to be good for the stomach, and they go to church to be baptized and to drink tsebel. My mother is very religious. She goes to church. She drinks holy water, *tsebel*. Sometimes she gets baptized with it. I think it's because they believe in their religion that it will cure them. I don't know; but when I think about it, I believe this illness is related to the Creator. This illness itself leads to religion, to something spiritual. When we talk to other patients at the hospital, they say that this illness requires *tsebel* treatment. That's when I started to believe.

**Participant 2 women caring for aunt diagnosed with cervical cancer, Addis Ababa, Orthodox Christian**

**I am caring for my aunt who was diagnosed with cervical cancer. She had no previous illnesses; this was also an incident for us. Since she did not have children, she was taking medicine for things she did to give birth; maybe that was the reason. I only found out now when she talked to the doctor.**

**She can't get bathed in holy water, Tsebel, now, but she drinks it. It is your faith that saves; by the power of God, she is now getting stronger. It comforts her to do it. You must know that you can overcome anything by faith.**

**But I believe that it is necessary to undergo biomedical treatment; doctors are given to us by God. It is good to pursue your medical treatment while exercising your faith. God did not say not to do treatment; I do not think that avoiding medicine is a solution. I think that all diseases come from Satan. But everything is God's will. And because God allowed it, she was given medicine, so she had to take it.**

**I've been to many places to get Tsebel; many times you turn to God in times of trial or when you're in trouble. I'm not the kind of person who goes to church often; when you are in need , you go wherever you can get help. There is a church in the Tafo area; many cancer patients are cured there, and so I went there and brought Tsebel.**

**Participant 3 men caring for his daughter diagnosed with childhood cancer (kidney cancer), girl, 6, Muslim**

I am caring for my daughter who is suffering from kidney cancer.

When I heard that it was cancer, I was shocked and said, "O Allah, save me, it is all in your hands, make it easy for me, there is nothing that is too difficult for you; make it easy for me."

I do not see cancer as Allah’s punishment; it is a test of faith that He (Allah) brought into our family, but not because of our sins or our wrongdoings. It is something Allah wants to see how we respond in our faith during suffering. We have no choices other than to accept it and stay faithful in our prayer for His forgiveness and help.

So when it comes, we accepted it and pray saying, "Allah, you save me, you help me."

**Participant 4, father caring for son diagnosed with Leukemia, Muslim, from Dessie, Amhara region**

There is a traditional medicine our ancestors and mothers used, called Feto. It's believed to help with healing. We applied some Feto, but it didn’t seem to make a difference. The woman we consulted was a traditional healer, and many people say they’ve been cured by her treatments. When we showed her the symptoms, she said, “This is that kind of disease and this is the medicine for it.”

But we chose not to use it. We thanked her and went straight to the healthcare centers instead.

She believed the illness was beyond the reach of conventional medicine. According to her, the cure required applying Feto to the skin of a sheep or using the skin of a black cumin plant. That, she said, was the traditional way to treat it. From her experience, she was certain there wouldn’t be a medical cure for this disease. Still, we didn’t give her the chance to try her method. We wanted both options investigated side by side—but the illness continued to worsen, week after week.

We’ve reached a point where we’re open to trying it. Let’s see it through to the end—maybe there is something, a treatment that can stop the root cause. Perhaps something exists that can regulate or stop the abnormal behavior of the white blood cells. We don’t believe it was cancer.

Personally, I think cancer is something related to genetic. I also believe that packaged and processed foods have a huge role in it. That’s why I don’t allow my children to consume too many processed foods. I avoid them myself. I don’t like them.

This illness isn’t related to religion. It’s been given an old name, likely because there’s been no proper research into it. But today, with technology guiding our lifestyles and diets, we’ve moved far away from natural foods—and that, I believe, has increased the risk of illness. Chemical-laden foods have played a major part.

Still, our priority is to save the child. So we turn to the Lord, Allah, and we ask for His forgiveness.

Sometimes, people don’t really understand how close illness can come, simply because they haven’t experienced it personally. They think, “It won’t happen to me.” But it’s natural—it can happen to anyone. Allah allows things to happen for a reason.

But I don’t believe that illness with cancer is punishment. I don’t think the way people used to in the past. We need to look forward—with faith, knowledge, and responsibility.

**Participant 5, father caregiver for his daughter, 12, from Dessie, Amhara region, Orthodox Christian**

I still don’t know whether the cause was hereditary or something environmental—maybe something that happened while she was playing. She used to bump into me while playing football. Later, we were told that the tumor had already spread and was beyond our control. It’s been about five months since then.

My wife has diabetes, but not cancer. I don’t think our daughter’s illness is connected to that. But my wife once told me about a dream she had. In the dream, she saw that taking a healthy, active child—one who moves around like a baby—and making her sicker was like taking away her light. It was just a dream, but it left an impression.

We believe that God created science, and for that reason, there is no excuse not to seek medical treatment. Treatment should always be the first step, even if you turn to spiritual practices as well. Faith and medicine can go hand in hand.

Personally, I don’t believe this illness came from God as a punishment. I believe it may be the result of environmental factors. Some say it could be something traditional or even spiritual, but I don’t believe it’s a judgment from God because of sin.

I didn’t try every option. I had a younger brother who passed away from HIV. He was a bright student—he graduated second in his class from Bahir Dar University. He was deeply religious, and in the end, he put all his faith in God. He used to say, “Let’s go to Tsebel” (holy water). Even though he had a serious illness, he chose his faith and believed in it fully.

But I believe that God also created medicine for us to use. I haven’t gone to Tsebel yet myself—but I don’t rule it out either. My belief is that both science and faith have their place, and we should pursue both when facing something this serious.

**Participant 6 father caregiver for his son, 11, diagnosed with childhood cancer, Muslim**

I believe that this illness is the work of the Lord, Allah.
Perhaps it has come to punish us because of our sins; but children do not know anything about this matter; it is not their sins. My children live well and carefully.
We did not try traditional medicine. We did not try to go to a religious place seeking spiritual help.
We are Muslims. We took him directly to the healthcare center seeking medical treatment.

**Participant 7, father, caregiver for his son diagnosed with childhood cancer, Muslim**

Since the onset of the symptom, I took my son straight to the doctor. We didn't try any other treatment, like traditional medicine, nothing at all.

First, I took him to the health center, then to the hospital; then we were referred to the tertiary hospital, alert hospital.

Cancer was brought by Allah. Is this something caused by humans? it could be that Allah is angry with me. It is Allah's anger. It could be to test me. It could be to bring me closer to Him. It could be to bring me closer to Him or to make His way clear. Maybe it's His way of correcting my path, bringing me closer to Him.

Cancer is a very serious disease. I don't think that the solution will come from humans or lies with doctors, but from Allah's decree. May Allah decree his mercy and give the doctors His wisdom and let them work on it.

**Participant 8 father caregiver for his son diagnosed childhood cancer (Leukemia)**

I don't know exactly what causes cancer. I don't believe in the wrath of God.

Some people in our community said it might be caused by the Evil Eye or demonic influence. When evil forces remain on a person for too long, it is believed to turn into cancer. We were told that if a person is attacked by evil spirit and not treated in time, it can eventually mark as a physical illness such as cancer.

Rather, it could be hereditary, or it could be caused by the evil eye. Nowadays, evil spirit is sent to make a person sick. Personally, I even thought of going to church to bathe in holy water. I had that in mind. God is the source of wisdom. So I believe He can guide the treatment too.

I don't believe that it is only by faith alone that He helps; God helps your path, He provides a person (doctors), He clears the way for you, and He makes it easier. So I believe in biomedical treatment and spiritual interventions.

Once, when we were going to holy places called Bole Arabsa Sefera to bathe in and drink the holy water, it was difficult for him to walk because it was a long walk. As a result, we couldn't do it again because it was too difficult for me. I believe in both medical treatment and spiritual support. I believe this, too, helps with healing. When the spirit is humbled or broken, God's help enters. I believe the spiritual plays a role for those who are willing to put in the effort themselves. It can guide you, support you, and carry you through everything.

**Participant 9, young woman, mother caring for her daughter diagnosed with leukemia, Muslim**

I believe that cancer is the wrath of God. It is our sins that brought it upon us.

Some say this is the wrath of the Creator.
This is a result of weakness, of turning away from the right path.
I ask myself, what did I do wrong?
Everyone makes mistakes, we are only human, but still, I wonder: What have I done to deserve this?

People also say that the God tests those He loves.

Some even believe cancer comes from marrying outside your ethnicity, or from marrying a close relative.
Maybe that’s why people distance themselves. For example, in my case, from the beginning my parents did not accept my marriage because my husband was not from our ethnic group. Some members of my family disapproved of our union, questioning my decision with remarks such as, ‘Why did you marry an [ethnic group]?’ When my daughter was later diagnosed with leukemia, they said it was a punishment for going against their decision. In their eyes, my daughter's illness was not just a disease, but the consequence of my transgression of family and cultural norms. To be honest, I also initially believed that my daughter’s cancer was somehow linked to the ethnic differences between my husband and me. A few even went so far as to suggest that the illness itself was a form of punishment for our interethnic union—as though our marriage had invited this misfortune. As a result, I have no one from my family to support except my uncle, who checks on me sometimes

**Participant 10, woman caring for her mother diagnosed breast cancer, Addis Ababa, Orthodox Christian**

Many people believe cancer comes from packaged products—and honestly, I used to think so too. Those things have chemicals, and chemicals have side effects.

We go to religious places. Especially after chemotherapy, Tsebel or holy water is a must. Now even she came here after drinking Tsebel.

Still, I believe this: **God does what He wills.** He is the one who gives knowledge. He will forgive you as He wills, whether through Tsebel or through treatment. He will forgive you as He wills. Mercy is from Him. He forgives as He chooses.

I don't believe that cancer is the wrath of God; I really don’t. I do believe that it is the work of Satan. I believe that all diseases are the work of Satan, but, if it reaches you, and if you die from it—then that, I believe, is from God. Because death itself is part of God’s plan. And God is not an angry God. If He were, who among us could escape His wrath?

But society thinks that the wrath of God has decreed on us.

I really pray. And I believe God hears. I believe that He will hear. If you pray with a sincere heart, He listens. I didn’t even pray that she wouldn’t get sick— I just prayed, “Please, don’t let her die.” That’s what I asked for. And I believe in the power of that prayer. We spent hours praying at home. And our tears—those tears reached God. If you had asked me back then what I believed about cancer, I would’ve answered very differently.

**Participant 11, men caring for his wife diagnosed with lung cancer**

I don’t fully understand how cancer happened.
I don’t have deep knowledge about it, and I never gave it a reason—because it just happened.
It's difficult to label something like this.
I don’t believe the God brings disease to destroy human beings.

I don’t know exactly how it started, but it could be related to many things.
For example, after arriving here, I heard that it might come from an untreated wound that didn’t heal properly.
That was the first time I heard something like that.
Before, I never imagined that something like this could happen because of that.

And I don’t believe it came from God.

That’s why I reject the idea that “God punishes with this disease.”
To me, that’s just something people say.
I don’t accept it.

My religion is Orthodox Christian.
And in the end, we go to Him (God)—who else do we have besides the Creator?

I will go religious holy places. I have faith.
But my wife didn’t try in relation to the illness. She didn’t seek help or healing from that perspective.

**Participant 12 men caring for his mother diagnosed with cervical cancer, from dessie, Amhara region, Orthodox Christian**

From what I’ve read, cervical cancer is more likely to develop after menopause.
I’ve learned that these tumors often grow slowly over time and can eventually become cancerous.
That’s why I believe it’s always important to get regular check-ups—so treatment can start early, before the illness becomes too serious.

And even with that, I don’t see any connection between this disease and the wrath of the God.
We’re educated people; we understand that.
In fact, I think she should be paying more attention to other possible illnesses as well.
This might have happened simply because it wasn’t detected early enough.

But once cancer is present, you must pray—while continuing treatment.
We pray, and she tries to pray alongside the medical care.
She doesn’t go to church often, but she does come sometimes.
It’s good to do both together—spiritual support and medical treatment.
I believe prayer strengthens the treatment itself.

Psychologically, it makes a big difference.
If you do nothing, you may start to feel hopeless.
But if you take action, if you pray and follow treatment, you stay hopeful—and that hope helps you heal.
I really believe the psychological side is powerful.

**Participant 13 women caring for her mother diagnosed with breast cancer, Addis Ababa, Orthodox Christian**

What kind of anger is this? God, how are You punishing me? Why? Why are You punishing my mother like this, who spent her whole life as a devoted Christian and is so committed to her beliefs? What sins have we committed? It is very difficult. I don't know; I've been in conflict with God. Why is she being punished at this age? Seeing her like this breaks my heart. Why do you allow it, God? It’s unbearable to face such trials in the later years of life.

This truly feels like the wrath of the Creator—what else could it be? Maybe even the wrath of Satan. She was always a woman full of energy, always moving, never stopping… and now, to see her like this—it’s heartbreaking.

My mother used to tell me to take her to the hospital. I was the one who insisted, who pushed her to get treatment. And sometimes, when she was angry or upset, she’d say, “You’re the one who brought me into this.”

I usually pray, “Oh God, please spare my mother. She once said she was healed because of me.” But I dragged her into it… she said it was the hospital’s doing. And I know—healing doesn’t come from the hospital alone. The doctors are just people. A hospital can help, but it doesn’t guarantee a cure.

It hurts. It feels like such a heavy burden… and such a shame.

**Participant 14, young man caring for his father diagnosed with cancer, Addis Ababa, Orthodox Christian**

I think cancer might be connected to what my father eats and drinks, especially since he spends a lot of time out in the fields.

He doesn’t go often to church on his own, but we take him when it feels necessary—when we really feel the need to pray or seek blessings.

**Participant 15, men caring for his sister diagnosed breast cancer, Orthodox Christian**

Cancer is the work of Satan, what else? In the past, HIV was the fear, but now cancer has become a very serious problem.

**Participant 16 woman caring for her grandmother diagnosed sarcoma, Addis Ababa, Orthodox Christian**

It’s a very unpleasant experience—something that’s hard to even put into words. People tell you not to cry, not to get angry, but when you’re in the middle of it, how can you not feel those things? Holding back emotions can sometimes lead to a loss of faith. You start questioning everything—your beliefs, your strength, your family, even God.

Why did this happen? Why us? It can create conflict with your belief. It's a disgusting, painful situation—emotionally draining. And yes, it's deeply unpleasant.
In some ways, yes I associate cancer with religious explanations. We lived in Harar and came to Addis Ababa after our father passed away. After that, because there was a series of deaths in our family, I started asking why this happened to us. Now, when something goes wrong or when there is no happiness at home, I often ask why, but not about the illness. It is an effort to understand the meaning behind our suffering and losses. But as time passes, and sadness becomes more familiar, you stop asking. You stop expecting happiness, and you just try to live through it.

When it comes to illness—especially something like cancer—we often feel helpless. Since there’s no clear solution, I ask myself: why don’t we try to treat ourselves in some way? I used to be so sure it would never happen to me. But that’s foolish, isn’t it? The truth is, we’ll never fully understand the reasons behind it. Maybe our bodies are always trying to heal, and eventually… they just can’t anymore.

That’s why I wonder—why don’t we support our bodies more? Maybe what we really need is better nutrition. Right now, I’ve been giving her vegetables, greens, trying to support her health naturally. Honestly, I don't think most of us think deeply about our diet—we just eat what’s available. But if we approached treatment with intention, I believe we could make a difference, even if it’s small.

We may not know the outcome, but at least by helping our bodies, we give ourselves a chance. A doctor even told me he used herbal medicine. That stuck with me. Sometimes, the medical system doesn’t offer all the answers. So why not try what we can? Why not help our bodies while we still can?

I’m not focused on what *caused* the cancer anymore. I’m focused on what can help. What can heal. That’s where my hope lives now.

**Participant 17, men caring for his mother diagnosed with breast cancer, Dessie, Amhara region, Orthodox Christian**

Cancer is an incident—something that can happen to anyone, at any time. Whether it's due to genetics or environmental factors, both play a role. We're human, and part of being human is facing the reality that illness and eventually death are inevitable. As we age, our risk for diseases like cancer or heart conditions naturally increases. Simply by being human, we carry a higher likelihood of developing conditions like cancer over time.

**Participant 18 men caring for his friend diagnosed with liver cancer, Addis Ababa, Orthodox Christian**

We know that cancer is a serious and often deadly disease. It has different stages, and I’ve even bought a book to learn more about it. It’s a terrible illness. I don't know exactly where it comes from, but I understand that it needs to be treated with care and attention. Doctors say that from stage one to three, there’s still hope. If it’s caught early—before it spreads—there’s a chance of survival. I know someone who’s lived for twenty years after being diagnosed. With proper treatment, self-care, and support, it’s possible to live a long life.

There’s a common saying that cancer is "God’s wrath," but I think that’s more of a traditional belief. For example, I have a cousin with seven children who refuses treatment when we try to convince him. There was also someone who threw away their medicine and went to Debre Berhan seeking spiritual healing, but sadly, he passed away just a month after going there. So, while belief and faith are powerful, I believe it’s possible to combine spiritual practices—like using *tsebel* (holy water)—with modern medicine and science.

Some doctors also believe in traditional or spiritual healing. I do too. I believe in the power of healing. After all, many modern medicines originally come from plants. Science has just developed and refined them. But in our culture, there’s sometimes a superstition: when people stop taking their medicine, they deteriorate quickly. I’ve seen that happen.

My friends also remind me that it’s God who gives people the wisdom to create medicine. All wisdom comes from God—including medical knowledge. Medicine, in its truest sense, is a gift from God.

**Participant 19 men caring for aunt diagnosed with breast cancer, from Oromia region, Orthodox Christian**

…There’s a clinic back home where she used to go for these injections. After getting the shot, they would let her rest for a while, and eventually, she would feel better—that’s how she managed for some time. But she didn’t seek help elsewhere; she didn’t go to tsebel (holy water). Now, she came here specifically for the *tsebel.*

Later, we went to the hospital to get medical treatment. That’s when we decided to really start fighting the illness. But when things got worse, we had to return to the hospital again.

I think it might have been caused by the food—my aunt was sick and weak, and I believe that contributed to it. Still, I don’t fully understand why it happened the way it did. Sometimes, I feel like it’s the work of Satan.

**Participant 20 women caring for her father diagnosed with prostate cancer, Orthodox Christian**

You know what happened to my father—he wasn’t treated in time, and that’s why everything unfolded the way it did. From what I’ve read and heard, prostate cancer typically affects men over the age of 45. If a tumor is found early and removed, the chances of it turning into cancer are much lower. But in his case, it wasn’t caught in time. Two years is a long time—the tumor kept growing, blocking his urine. Something was happening inside, and eventually, it developed into cancer. We repeatedly urged him to get treatment, but he kept working. He insisted he wouldn’t stop working, even as he kept getting sick. That’s why it progressed—he wasn’t treated in time.

We are all Orthodox Christians. I personally bathe with Tsebel (holy water) in faith. From a holy place called Abune Hara, “I brought tsebel and faith and gave it to our father,” because we had heard that Tsebel could help cure cancer. But I didn’t have him bathed in the holy water itself, because in my opinion, it didn’t feel right—especially with stopping chemotherapy and turning entirely to that. I believe that faith is something in the heart. He drinks Tsebel at home. We honor his faith—he prays, drinks the holy water, and does everything he can, except actually being bathed at the site. Our religious Father prays for him, and we all pray. But I held back from bathing him in Tsebel because I was worried—the cold weather had weakened him, and he had no strength. Some people give up treatment completely, turn only to faith, and then come back even sicker. That’s why I said no to the bathing in holy water—but we keep praying, using holy anointing, and holding onto faith inside the home.

Sometimes our neighbors had also brought us a plant they said could cure cancer. It was a type of green leaf. We were told to drink it like tea, morning and night, without sugar. We learned about it ourselves, searching YouTube and Google. It’s a round leaf, crushed into a tea—something like moringa. I saw people online talking about it, even showing blood samples and saying it worked. The medical process we saw at Tiku Anbessa Hospital looked just like what I saw online, and I found that encouraging. I didn’t just rely on one source—I compared two or three different ones before trying it. The leaves came from far away. I even drank it myself, but honestly, I didn’t see much difference.

Abune Hara is a holy place, and faith in Tsebel is being promoted heavily on social media. We saw posts saying it cures cancer, HIV—many illnesses. There are people who say they’ve been healed through faith and Tsebel. It’s not just about the water—it’s about drinking it with true belief. That’s what people online were saying: “Go to Abune Hara Dengel.” So we thought, why not try it? We believed, we drank the Tsebel, and we held onto hope.

**Participant 21 men caring for his daughter diagnosed with Leukemia**

We’re still trying to understand the cause. It could be related to her diet or possibly even a physical injury, like a fall—she had a history of abdominal trauma. But we can’t say for certain that’s what caused it. There are many possibilities, like environmental exposure to chemicals or even hereditary factors. Still, we can’t pinpoint one specific cause.

For instance, there’s a gold mining factory just six kilometers from where we live. The water in the area has become contaminated—animals were drinking water tainted with acid used in the gold refining process. We lived near that area too, so I can’t rule out its effects, but again, it’s not something we can say for sure without proper investigation.

In our community, people often advise against relying on too many people or institutions. They tell us to go to churches, prayer houses, or try traditional medicine. Personally, I believe if there’s something in our diet or in traditional remedies that helps—like abash or black cumin—we’ll use it. These are natural spices and harmless when used properly. We use them.

However, there are some who discourage going to hospitals altogether. They say, “If you go to the doctor, you’ll die.” That kind of thinking still exists in the community. But for me, faith and medicine go hand in hand.

Religion is deeply personal—your faith is what guides you. We believe in a Creator. We pray, and we know it is God who ultimately heals. A doctor can treat, and we can pray, but it’s God who decides the outcome. If He chooses to heal, we’ll see signs—some immediate, some gradual. Right now, I’m praying that the medicine will work, and others in our community are praying with us. We go to church, and others—like our Muslim neighbors—pray in their own way, even in open fields. We all believe in God. We believe He is with us and that He will save us.

God has always sustained us. He gives us life, breath, the sun, the rain—things no human being can provide. Without oxygen for even a few minutes, life ends. So we thank Him and continue to pray that He will save us. But we also understand that God doesn’t only work through miracles—He works through knowledge, through medicine, and through wisdom. The medicines we take are made from the very creation He gave us. So we will continue to pray while also pursuing medical treatment.

As a professional myself, I believe in using every option available. Modern medicine is still relatively new when compared to traditional practices. In the past, people used traditional remedies. But we have to ask—how does traditional medicine diagnose cancer? How does it determine if a treatment works? Without evidence, without scientific validation, we can’t be sure. That’s why confirmation is necessary: understanding the pathology, how cancer cells behave, and how treatments target them. As professionals, we understand these systems, but many in our community lack this education, so it’s not surprising that they rely on what they know and believe.

**Participant 22 women caring for her father diagnose with prostate cancer, Orthodox Christian**

Cancer is a very frightening disease and something that demands caution. Personally, it’s made me more aware and reflective. I’ve started thinking more seriously about my lifestyle—especially my diet. I’ve come to believe that packaged foods might be linked to cancer.

I’ve read about it in different places, and while it’s not always easy to avoid packaged foods, I believe they can contribute to the risk. That idea has stuck with me, and now I try to be more careful about what I eat.

We’ve considered religious methods. But my father doesn’t have very strong faith, so that path hasn’t been a major focus for him. Still, we’re doing what we can—both medically and spiritually—wherever possible.

**Participant 23, women caring for her father diagnosed prostate cancer, Debre Brihan, Amhara region, Orthodox Christian**

My father has no interest of going to church or religious places. In the past, he wouldn’t miss church and always prayed regularly, but since his illness, he has stopped going as often. I think the illness has affected his routine and motivation.

Some people go to religious places believing the holy water (tsebel) will heal them.

My father isn’t very interested in tsebel. His sister brought it to him once, and he drank it out of respect, but he wouldn’t go seek it out himself. He has no personal experience with that kind of spiritual practice.

He believes more in medicine. If he’s given a treatment and told it will help him, and he sees improvement, he immediately believes in its power. That’s where his trust lies—mostly in medical solutions.

**Participant 24 women caring for her father diagnosed with prostate cancer, Addis Ababa, Orthodox Christian**

The name "cancer" itself is very scary and requires caution. I myself am now thinking a lot about it; I have realized that I need to be careful with packaged foods. I think that packaged food causes cancer. I have also read that consuming packaged items is very bad for health.

My father is not very strong in religious matters. We did not delve much into religious issues related to our father's illness. When our father was sick, we went directly to the doctor.

**Participant 24 women caring for her son diagnosed with brain tumor, Benshangul Gumuz region, Protestant**

I thought cancer was the wrath of God at first. I always questioned myself what sin I had committed that was different from other people. When I went to the hospital, I would ask myself, how did this happen to me? When I came back to my senses, I would say, I did not fight with the Creator. Thank God, there are those who have survived cancer, as I have seen here. But most visit the hospital at an advanced stage. So God is the one who gives life; we try, but it is only God who gives life.

During the diagnosis, I frequently questioned what sin I had committed that this anger and condemnation came to me. But later, I recalled a story of a man who was born blind since childhood from the Bible. In this story, people gathered and asked Christ whether the man's blindness was because of his mother's or father's sins. But Jesus Christ answered that neither of his parents had sinned; it was so that the glory of God could be revealed. That gives peace of mind. We are all sinners while on earth; if he says, "I am righteous," he is a liar. So, before we came here for his treatment, I am a Protestant. I went to the church before starting anything; then people prayed for my son, and we were sent for treatment. Thank God, God is with us; people are not only praying for my son, but we are also praying for the doctor who is treating him. Because the doctor is a man, he may use his knowledge, but it is not the doctor who saves the soul; it is God, so we prayed first.

I don’t support the idea of stopping medical treatment and relying only on prayer, because as written in the Bible in the Gospel of Luke, Luke is a doctor; he used to give medicine. So before you go to the doctor, there is a God whom we should consult. It is God who put Adam himself in a deep sleep and first surgery on him; so God performs the surgery, and the doctor who gives medicine is Luke. Therefore, it is possible to combine faith and treatment, but many people stop and go to religious places seeking miraculous healing. I do not believe in that.

Yes, confusion is part of the journey. But God didn’t bring me here to make me angry or to break me. He brought me here to show me something greater. I’ve come to understand this. And now, I sit in peace. I am grateful to God.

When God allows something to happen in our lives, it’s not to destroy us or defeat us. It’s to reveal something deeper. And in that, I find strength.

**Participant 25, man caring for his daughter, 11, diagnosed with kidney cancer, from Sothern Ethiopia, Protestant**

After she started treatment, you took my daughter to a place of prayer; especially before she had surgery. After I put my daughter in the hospital, I personally went to the place, where there’s a private prayer; but my daughter didn’t go in person—I went on her behalf. I stayed only for that night, and after the prayer I came back to the hospital.

**Participant 26 women caring for her daughter, 3, diagnosed with leukemia, Dessie, Amhara region, Muslim**

What we used to hear about the cause of cancer was that it might be related to nutrition or poor diet, or that some kind of issue with food could cause problems.

But I never fully believed that explanation. My faith was in my Creator. I never thought something like this could happen—not to me, not in this way.

I mean, I never thought God would allow something like this to happen to my child. It never crossed my mind that this kind of suffering would come upon my child.
In fact, I believed that even if hardship came, God would make it light, easy to bear. That was my expectation.

I don’t believe cancer is a punishment from God, not at all. I believe it's from the devil. But of course, even then, nothing happens without God's permission.
Sometimes He allows it for a reason. It all comes through His command, even when it's the devil that brings it.

Honestly, I don’t know. I can't say for certain where it came from—whether it's from God or not. I really don’t know. But I believe that nothing happens without God’s permission. Maybe it's just part of the journey, going in circles, as He allows. But how or why—only He knows.

I am not living with my husband and we’ve been separated for some time. Also, my mother had cancer in the past, so his family thinks this illness is something that runs in the bloodline. Because of that belief, they don’t even call, and no one from his side offers support.

They (my in-laws) assume it's hereditary, they don't show concern, they don’t ask about us, and they don't try to help. They know everything—the diagnosis, the stage of the illness, all of it. But if they don’t want to help, I can’t force them. So I stay silent. I don't say much anymore.

Their attitude doesn’t affect me anymore, because I believe I am fully responsible for my child, and that responsibility is mine alone.

As I mentioned, he and his family seem to believe the cancer is genetic and that I brought it into their lives. So they keep their distance. They assume she might not survive.
Their questions are never helpful—they ask if she’s going to live or die, as though it’s just a matter of outcome. That hurts me. They don't ask how they can help.

Sometimes, I don’t even want to answer their calls. Only God knows whether she’ll make it or not—not them. Repeated questions like, *"Will she survive?"* weigh heavily on me. And since they don’t offer support, I see no reason to stay in constant contact. That’s why I often ignore their calls.

**Participant 27, woman caring for her sister diagnosed with kidney cancer, Oromia region, Muslim**

At first, when we were told it was cancer, we stopped the medication. We thought maybe it wasn’t something natural—we believed it might be spiritual. We considered it could be due to the "evil eye," or maybe even a demonic influence. Because of that, we chose to approach it through faith instead of medicine.

We started using holy water from a Qur’an house, and prayed a lot. We thought spiritual intervention was the way to go. So initially, we didn’t commit to medical treatment—we believed it was something that had to be addressed spiritually.

We had been told by doctors that the Tumor was cancer, and they recommended starting treatment immediately. But we didn’t begin right away. We diverted our path to traditional and spiritual practices. We tried faith-based healing—holy water, prayer, and other spiritual means—before we ever started any chemotherapy or hospital treatment.

Yes, we wanted to understand the root of it. But we never thought of it as God’s punishment. Instead, people around us suggested it might be from the evil eye or demonic influence—that when such things remain on a person too long, they can turn into cancer. That’s what we were told by people in our community.
They said, "If a person is spiritually attacked, and it's not addressed, it can transform into a physical illness like cancer."

Because chemotherapy is known to be very harsh, we thought it would be better to first pursue healing through our faith. Yes, a lot of people around us think that way. It’s a common belief.

Now, everything is much clearer to me. I understand that cancer is a normal, medical illness. In the beginning, I really did think it came from a spiritual issue—maybe the evil eye that turned into cancer.
But now, after seeing the effects of proper treatment, and how her condition changed with medication, I know it’s a real physical disease—something that needs medical care. I’ve come to accept that.

**Patient side interviews**

**Participant 29 men diagnosed with cancer, Oromia region, Protestant Christian**

When I heard my diagnosis for the first time, I said to myself, “If this is from God, then so be it.” At first, I thought, “No, it can’t be,” but then again, I accepted—“If it is, then it’s what God has allowed.” That was my understanding.

Now, I understand that when something like this happens, you have to both pray and seek medical treatment. That’s the message I’ve come to accept: rely on God, but also go to the hospital and take the medication—because healing can come through both faith and medicine.

**Participant 30, women diagnosed with cervical cancer, from shewa robit, Amhara region, Muslim**

For some times, I felt my diagnosis as God's punishment for my sins or wrongdoings. But, over the course of the illness, I came across to see it as a test of faith. I do not see my health situation is the result of God’s punishment rather I accept it as how God wanted to see my faithfulness and how I endure despite suffering.

**Participant 31 women diagnosed with breast cancer, Oromia region, Muslim**

It has been three months since my breast cancer diagnosis was confirmed. In the beginning, I was so saddened when I heard a cancer diagnosis was confirmed thinking about the future of my children, and questioned Allah seeing it as a punishment, but later I come to a knowledge that it might happen because He (Allah) saw something good in me. Islam teaches that something might happen for a divine reason, if not, it might not have happened to me.

**Participant 32 woman diagnosed colon cancer from Addis Ababa, Orthodox Christian**

Besides following my medical treatment, I do use *Tsebel* (holy water) and rely on my faith. That has always been a part of my life. Because of my religious beliefs and because there’s a church nearby where I can access holy water, I often go, pray, and drink the holy water. It’s something I do regularly—not because I expect it to cure the illness directly, but because it gives me peace and helps me feel stronger.

I also take communion and participate in church practices. That’s my way of finding spiritual satisfaction and comfort. It’s not that I’ve relied on herbal medicine or traditional remedies—I haven’t taken those. But when I feel symptoms like dryness or discomfort, I might drink ginger tea, fenugreek, or other simple natural drinks—not to treat cancer directly, but to ease how I feel at the moment.

From what I’ve learned, every person has cancer cells in their body. It’s just a matter of when the condition gets triggered and where in the body it manifests. There are many things that might contribute—our diet, how we live, stress, and emotional strain. So I think the cause could vary from person to person.

In my case, I don’t think I had particularly unhealthy habits. But looking back, I realize I ignored certain symptoms—especially dryness—and didn’t seek care right away. For example, I used to eat food and feel something strange, but I wouldn’t address it. I would just tell myself, “I’ll rinse my mouth tomorrow,” and keep putting it off.

I believe that lack of attention and not acting early made things worse. If I had gone to a hospital sooner, maybe they could have found a solution earlier.

Still, I believe that no one dies before their time. Whatever I try, if my time has come, it has come. But I do pray to live without suffering. I have no idea what brought cancer into my life. But I do have a feeling that I may have done something wrong in my life; related to living in accordance with fully obeying God. Perhaps it was because I was unable to live according to His will. Perhaps it was because of this that I suffered the wrath of the Creator, but I understand now that it can happen to anyone. And maybe it happened to me so I could learn something meaningful in my life. That’s how I’ve come to see it—not in fear, but with a sense of purpose.

**Participant 33, men caring for his father diagnosed with liver and colon cancer from Addis Ababa, Protestant**

Even before this happened to me, my mother had been ill for a long time. Since I cared for her and saw how long she relied on medication, I used to suspect that such long-term use might lead to complications. I used to say the same to my father as well—that it could have long-term effects, and I tried to mentally prepare myself for such possibilities. I believe in being proactive and preparing ourselves for such situations, including emotionally.

What I’ve come to understand is that medicine, while helpful, can also create its own problems if taken indefinitely. We sometimes take medications for everything without considering the long-term consequences.

Yes, I’ve had that habit since I was young. I used to get sick often, and I’ve always believed that proper healthcare includes taking personal responsibility. For example, I suffered from tonsillitis for years, and eventually a doctor told me, “Treat yourself—adjust your lifestyle.” That experience taught me that while medicine helps, we also need to make lifestyle changes.

Some health problems don’t just come from disease—they come from how we live. For instance, someone who likes salty food might need to reduce their salt intake. In my case, I used to eat very spicy food, but I’ve stopped now. I’ve learned to take better care of myself, and I’ve been healthy for the past four or five years.

So yes, I trust in medical treatment, but I also believe in being responsible for my own health—by being cautious and living wisely.

**Participant 34 men diagnosed with liver and colon cancer from Addis Ababa, Protestant**

I accept what I have because it is brought by God.

I accepted it. If you cannot fight it, then you must accept it. I cannot break down this wall; my only option is to face it and endure. I accepted it because nothing is above God. I also decided that I must take the necessary treatment.

I don’t have any special opinion like some say it is God’s punishment or a test from Satan. For me, it is just a disease I am battling. I believe I am in God’s hands, and He will save me. My father used to be a health professional and owned a pharmacy. Because of my little knowledge about medicine, I trust that God is ultimately the one who heals, even though the medicine He helped creates. Wisdom and healing both come from God.

To be honest, I never had a clear understanding of cancer before. But looking back, I think it’s a condition that can develop when we don’t pay enough attention to our bodies over time. Now, I understand that cancer is a medical issue, and I believe treatment is necessary. At the same time, I also think medications have their own impact—especially when taken over a long period. In my view, using medicine continuously for more than a year can lead to side effects.

**Participant 35 women with colon cancer, Oromia region, Protestant Christian**

Even when we are in the hospital, we pray, we cry out to our Father, God. God has a reason. May God do as He wills. He will heal her. Our suffering is easy. He sent this to us for a purpose. This is our belief. Whenever God permits, He will heal. This is simple for us, and our burden feels lighter. Everything He does will happen at the right time.

We have never heard of anyone who has cancer. God also has a reason for the influence that Satan brings.

It is Satan who causes illness. God does not want you to suffer from disease. Instead of disease, I believe that God provides comfort and healing. We pray daily and cry, seeking His mercy and healing. God does not inflict suffering. However, Satan is the one who causes disease and suffering to family.

I believe God allows trials to strengthen us. It’s not meant to harm us through illness, but to heal us. I believe this. He will heal us quickly and lift us from our sickness. I have never been sick a day in my life.

**Religious Father: Orthodox Church**

Let me begin by introducing myself. I am a teacher by profession and I teach Dogmatic Theology at St. Selassie University. I also serve in the church, working actively in the congregation’s gospel ministry and in various other church activities. I am responsible for the gospel ministry in the Debre area.

When asked about the church’s teaching on health and illness, here is what I share:

I want to speak about the concept of illness. Illness can have different meanings. We—the church leaders—and the elders can provide explanations, but fundamentally, illness is something that is not natural to our human nature. It is something alien to who we truly are. Illness is like an enemy that harms, burdens, and destroys us. In other words, illness is not part of our natural constitution.

This understanding comes from Orthodox anthropology, which teaches how humans were created. According to the Bible (Psalm 139:14), humans are created in the image of God—“fearfully and wonderfully made.” This means humans were created as pure and good beings in God’s likeness. The Bible further explains (Genesis 2:7) how humans were formed, indicating that humans were created without sickness or disease; we were created as pure beings with perfect bodies.

**Teaching on Illness Based on Scripture and the Fathers’ Tradition**

When we discuss the teachings of the Church, grounded in the Holy Scriptures and the Fathers’ traditions, we understand that when a person is separated from God, God is life itself, and illness becomes a burden on that person. The saints’ writings testify that through Jesus Christ, God’s Son, human fallenness and destruction entered the world.

When asked how illness came about, the answer is that it is brought about by the person’s own actions. For example, a person may harm themselves through wrongdoing or neglect and thus become sick. Although there are many theories about illness, we can summarize the concept in four main parts.

Illness, or *dewe* (which means disease or sickness), can be understood and explained through four categories linked to biblical history.

Consider the story of a man who was bedridden for thirty-eight years, who lost his way, was far from healing, and remained without help—this is the example we speak about.

The first type of illness is called *Dewe ze-eset*. This refers to illnesses that have significant value or purpose. For instance, like Job’s suffering, who was without sin yet tested by Satan. The Bible explains that Job was righteous but was put to trial because Satan accused him before God, claiming Job would curse God if his blessings were taken away.

Despite his suffering, Job remained faithful to God, and God, in His wisdom, knew all and allowed the trial to teach and refine Job. Job endured great physical affliction and loss but kept his faith. Later, when he trusted God again, even after losing everything—including his wife—God restored all things to him.

**The Second Type:**
This is called *illness of oppression* or *possession*—an affliction brought by spiritual forces. For example, like King Saul’s case. Saul was hostile and troubled, and Scripture describes how David had mercy on him even as Saul was tormented (see the Book of Kings). Saul was in conflict with God, and David’s compassion shows that Saul’s affliction was a spiritual oppression. From the New Testament, we also see Herod’s example. According to Matthew 2:16, Herod, fearing the loss of his power, ordered the massacre of infants to try to eliminate Christ. This kind of illness is linked to spiritual attack and possession, where a person’s body is as if dead or controlled by evil forces. Such illness is called *illness of oppression*.

**The Third Type:**
This is *illness of purity and preservation*, called *illness of sanctity*. For example, Timothy suffered from illness, and Paul advised him to take a little wine for his stomach’s sake (1 Timothy 5:23). This illness does not come from sin or spiritual attack but rather from maintaining holiness and keeping one’s body from decay or harm. It is an illness that preserves the person’s sanctity, like a refining process. This kind of illness serves to guard purity, and we find it mentioned in the lives of Timothy, Paul, and Peter.

**The Fourth Type:**
This is *illness due to sin*—an illness brought about by wrongdoing or failure to obey God. It comes as a natural consequence of sin. For example, the Bible teaches that disobedience and sin can cause suffering or disease as a form of correction (see passages about punishment for sin). This illness calls the person to repentance and correction. When one fails to follow God’s commandments, this illness may come as a result.

Plague and sin

For example, Herod did not know that Christ was the Messiah; he was afraid that a greater king would arise. Therefore, what was commanded is a plague because it is unnecessary.

But if sin is not committed, the following is the law. He also said, "Do not sin; lest such a disease come upon you." This disease means that there is sin. It must be understood and acted upon as sin causes disease.

However, when we reflect on the matter, we cannot say that *every illness comes directly from God*. The reason is that there are diseases that come as a result of our own negligence or lifestyle. So, when trying to understand the cause, it's important to look at each case in relation to the individual’s life journey.

For example, a person who lives a promiscuous lifestyle might contract a sexually transmitted disease. But it would not be correct to conclude that every illness comes from one of four fixed causes. The Church does not teach that way either. To say that *all sickness comes from God* leads to the dangerous conclusion that God is cruel or harsh.

On the contrary, the Church teaches that God is kind, merciful, forgiving, compassionate, a Father, and capable of doing all things. His mercy and goodness are greater than even a mother’s love for her child. Because of this, the Church emphasizes God's rich grace and overflowing mercy.

At the same time, it is good to think about God's character holistically — that He is both just and merciful. What does that mean? It means He is full of forgiveness, and yet also a righteous judge. In other words, both aspects — His mercy and His justice — go hand in hand.

Still, God is a God whose mercy outweighs His anger. For example, the grace brought through Christ far surpasses the sin committed by Adam.

In the time of Noah, and in the days of Sodom and Gomorrah, we clearly see that God can become angry and that He judges. After teaching, warning, and giving people a chance to repent — and they still refused — He judged them in His wrath. We see how He destroyed Sodom and Gomorrah with fire.

However, to say that every disease that appears in different ways is a direct result of God’s wrath is too simplistic. That’s because illness can arise from many different causes. For example, even cancer cannot always be labeled as a consequence of sin — it's not that straightforward.

Rather, many diseases come as a result of how we live and what we do. God has given us nature — and when we misuse it or act against its design, there are consequences. Everything created has a purpose, and the way we use what’s created can have its own natural impact.

So, when people assume a certain sickness is a punishment from God, it might actually be more accurate to understand that they’ve placed themselves within the natural consequences tied to certain actions or choices.

Sometimes a person may or may not truly know themselves. Why? Because we are aware of the things we do — and since we know what we’ve done, we may say to others, *“God is dealing with me,”* believing He has turned against us due to our actions.

However, we must also consider whether our judgment is accurate. Is our way of measuring ourselves truly correct? A person might come to know whether what they have done is sin by comparing it with what is written in the Holy Scriptures or with the teachings of the Church. When one reflects deeply, they may realize how their actions oppose or contradict God's Word. They may recognize how God has long been patient with them.

One might even begin to think, *“God has tolerated me for a long time, and now He is punishing me,”* forming a personal conviction about their suffering. However, at the same time, it's also possible that this may remain unknown — because God's nature is not something we can fully measure or grasp. As Church teaching reminds us: there are things about God that can be known, and others that remain a mystery.

Therefore, since we cannot fully comprehend God's nature, we may struggle to clearly explain why a specific illness or hardship has come upon us.

That said, accepting everything with reverence and humility is essential. What does this mean? It means accepting that *“God is the one who does all things.”* When you accept that truth, it is a sign of faith. And in that faith, you will find freedom. God will give you peace.

Whether He acted or didn’t act, we trust in Him. If He didn’t deliver us, we still accept our suffering. It’s not only fire that tests a person; even sickness, in the moment it strikes, shows a person where they stand. Yet beyond that — the burden which surpasses our strength — that belongs only to the Master.

Why? Because I can only carry what I am capable of. But You, Lord, are the God who can carry everything — and I trust in You.

**Does modern medicine conflict with the healing ministry of faith?**

Personally, I don't see a major contradiction. When we talk about the Church and the coming of Christ, it's not only about spiritual healing. While some may consider Him as only a healer of the soul, Jesus actually came to save the whole person — both soul and body.

So when someone is sick, the Church offers healing. Why do I say that? Because the Bible clearly shows that Jesus healed many people suffering from various diseases — see Matthew 8 and 9. It says many sick people came to Him, and He healed them all. As the prophet Isaiah foretold in Isaiah 53:1, "He bore our sicknesses." This means that illness affects both soul and body, and both are addressed by Christ's work.

Therefore, the Church ministers not only to the soul, but also to the body. Jesus bore our weaknesses and carried our diseases. So, healing isn’t just symbolic or spiritual — it includes physical recovery. Through His word, He cast out spirits and healed all who were ill.

The Church, being the Body of Christ, carries this same power. Christ is the one who works through His Body. The same Jesus who healed yesterday still heals today — and He does so in the Church. She offers healing from all things — whether demonic oppression or physical illness.

The Church carries the burdens of people — both soul and body. And you can witness the healing of sick people, even those with medically diagnosed conditions, simply through anointing with holy oil. Many monasteries and churches provide this service, and countless testimonies of healing can be seen.

This doesn’t mean the oil contains some kind of chemical compound. The water or oil is simply united with God’s word and blessing — in the name of God, through the intercession of the saints, especially the Virgin Mary, and through the power of Christ, who is our healer. This is a trusted and historic teaching of the Church — that Christ heals not only our souls but also our bodies.

Moreover, His flesh and blood (in the Eucharist) are not subject to disease. That is why we call Jesus our Healer — not only from sin but from all things. So what I am trying to say is this: the Church offers healing that goes beyond hospital medicine.

Why? Because the Church is the true hospital — the house of God. Who is the doctor? Christ. Who delivers the healing? The priest, through the sacraments and prayers. The Church’s healing is greater than any other: it heals the body, heals the soul, and prepares us for eternal life.

Modern medicine may treat physical illness, and that is good. But what does the Church teach about modern medicine?

**The Church does not oppose modern medicine**. Rather, it embraces what is good and useful in it, while offering something deeper: healing that includes both body and soul — and that leads to salvation.

**Choosing Between Faith-Based Healing and Modern Medicine is a Personal Decision**

Going to a place of faith or seeking modern medical treatment is a personal decision for each individual. The Church does not teach that people should avoid hospitals. However, based on their faith, some individuals may choose to turn to religious healing instead.

Even in the Book of Sirach (also known as Ecclesiasticus), it is clearly stated that everyone should go to where healing is available and accept treatment. Nowhere does it say not to seek help.

At the same time, someone might say, “I won’t go to the hospital because Christ is both the medicine for my soul and my body.” That’s a personal choice too. After all, Jesus said, *“I am the way, the truth, and the life.”* He also said, *“I am the bread of life”* — but that doesn’t mean we stop eating actual bread. He said, *“I am the living water,”* but that doesn’t mean we never drink water again.

This shows that even if salvation is only through Christ, we still experience physical realities while on earth. That doesn't mean we are forbidden from using medicine.

If someone says, “Holy oil is what I choose,” and they believe with faith, they may be healed through that faith — just as Jesus said, *“Your faith has made you well.”*

Using medicine is **not** a sin.
Not using medicine is **also not** a sin.

For example, in my own life — I am 35 years old — I have never been hospitalized or had to visit a doctor. But that’s just my personal experience. People will experience things according to their own faith.

That does **not** mean people who go to the hospital lack faith or are sinners. No. Everything was created for good. We are taught to examine all things and hold on to what is good.

For instance, there are some medications that conflict with Christian principles — and I personally would not accept those. Even now, I cannot bring myself to take certain drugs that contradict my beliefs. But again, that does not mean using them is sinful — it just means I personally can’t reconcile it with my faith.

Still, we don’t teach or promote that people should rely *only* on medicine. We proclaim Christ, because He is the ultimate Healer of all things — both soul and body. That’s the Church’s position.

That said, as free and rational beings, everyone is entitled to choose according to their faith and understanding.

**Interview with Church Pastor context**

Differences in interpretation do not arise solely from levels of biblical knowledge or from what is written in the Bible. They are also shaped by the traditions within the Church and by our personal upbringing and experiences.

A person’s perspective on spiritual matters is not formed only by their faith. It is also influenced by their family environment, the kind of education they’ve received, the surrounding culture, and their personal experiences. All of these play a role in shaping how someone understands and interprets spiritual issues.

Therefore, in the Church, it’s not just a matter of *what* we believe, but also *why* we believe it. For example, if you visit different churches across Ethiopia, you’ll find that beliefs about sickness and medicine can vary widely. You won’t find a single, unified teaching that is agreed upon and clearly stated. Instead, you’ll encounter different explanations depending on the church or the teacher.

Still, as believers, there are some shared foundations we tend to agree on. For instance, when we discuss topics like **God’s anger** or **blessing**, people often start by referring to stories from the Old Testament. Those narratives are usually the first place people go to support their understanding of how God interacts with humanity.

In those Old Testament stories, we see God punishing His children who did not obey Him — and He did it in various ways. For instance, people suffered physical ailments, from visible skin diseases to conditions as serious as strokes. Take the story of Naval, for example: when David was told, "This is what should happen to you," he was shocked — and the text says God struck him, which some interpret as something like a stroke.

All of this is often understood in connection with God's anger. But in another sense, God also established standards for how people should live. He gave laws to Israel not just as religious commands, but also with practical implications — for instance, about cleanliness. He instructed them to keep their camp clean, even when they were in the wilderness, saying, "Because I, the Lord, will walk among you."

This shows us that modern scientific principles were already reflected in God’s commands. There were foods they were allowed to eat and others they were not — and God, being all-knowing, gave them these rules ahead of time. These dietary restrictions and cleanliness laws were not just about ritual purity but were also tied to health and safety. Especially when it came to certain kinds of meat, God gave His people clear guidance on what to consume and what to avoid, because He had chosen them and wanted them to live well.

What these things show us is that God taught His people to take care of their health. A person can become ill due to poor habits, or because they fail to maintain cleanliness. They can also suffer because of God’s anger. For example, in the Bible, there are people who were tested or tried—that is a key point. Take Job, for instance, who was tested. While the source of his trial is not directly God Himself, God sovereignly allowed it to happen, because nothing can occur without His permission.

After that, God arranged in various ways how He wants people to be disciplined and corrected, often allowing illnesses and sufferings as a form of punishment. For example, if a person shows some kind of physical symptom—like a rash, a sore, or a visible skin disease mentioned in the Bible—this was understood as a sign that either the person is unclean or that God is displeased with them. The first step was for the person to leave the community and stay isolated for seven days. Then the priest would come and examine them. If after the second examination the symptoms were gone, it showed that the illness was not caused by sin or God’s punishment. After that, the person would wash their clothes and be allowed back into the community. But if the condition continued, it meant the person was under God’s punishment.

Our Holy Scriptures also teach the use of medicine. For example, when Hezekiah was seriously ill, he used medicine on his leg. The Bible also talks about "gall" (a bitter substance), which was used as medicine; it explains that when the people suffered illness, the gall was the medicine that healed them. The gall is a place where medicine would settle and act to heal wounds. So, the Bible shows knowledge of healing and medicine and emphasizes the importance of prevention and cure.

When we come to the New Testament, there is no direct mention of such detailed regulations about illness or cleanliness like in the Old Testament. The transition period to the New Testament was complex and full of confusion. It was difficult to separate the old laws from new teachings.

The Bible also teaches that a person’s suffering is not always because of sin or punishment from God. Sometimes a person can suffer simply because of the brokenness of life or their own body, or even because of the sins of their family—though the Bible does not blame parents for all of a person’s troubles. Instead, it shows the greatness and glory of God even in suffering.

A person does not need a strict reason to believe in God. Sometimes, people suffer without understanding why. For example, the apostle Paul is believed to have had eye problems, according to historical sources. He often spoke about the struggles he faced, but also how God’s power was made perfect in weakness.

Paul explained that the hardships of the flesh, such as illnesses or temptations from Satan, were ongoing struggles. Some people interpret Paul’s words differently, but the main point remains: even among the apostles, there were those with medical knowledge. For instance, Luke, the physician, was among them. Timothy, another early Christian, had stomach problems and used remedies, including a certain type of wine, for his illness.

Paul did not forbid using medicine or remedies but encouraged proper care of the body. He also reminded Timothy to take care of himself.

In the New Testament, there is also a divine discipline. If people do not believe, God’s grace of discipline or correction would not be exercised within the church. This discipline can come in different ways. We believe it is part of a spiritual battle. Secondly, people may suffer because of carelessness or neglect. For example, Timothy is told that a little wine might be good for his stomach and other physical ailments.

The Bible also talks about proper conduct regarding diet; it encourages moderation in all things. Sometimes people cause problems for themselves by eating or drinking the wrong things, often because they don’t know the right measure. If they go beyond what is proper, they may fall ill.

The Holy Scriptures say to honor God by what you eat and drink in the name of Jesus Christ. Secondly, it warns against overeating or excess. For example, some who eat silently or without discernment do not realize that their bodies are temples of God and do not give proper care to their skin or health. God expects everything in moderation.

In this way, the Bible anticipates much of what modern science teaches today.

If you ask what our current experience is, we pray for those who are faithful. No matter what happens, our first choice is always to pray. We believe in a supernatural interference. Secondly, because God is loving and a giver of good things, I have faith that without God’s wisdom, doctors, medicines, and medical tools would not work effectively. Any wisdom that does not come from God is incomplete.

Any technology that benefits people and brings glory to God is His gift. This is because every good gift and every perfect blessing comes down from the Father of lights, the Creator in heaven. Therefore, we pray for those who are sick and also encourage them to seek medical treatment. Even when we pray, we do not discourage people from taking their medicine or following their doctor’s advice.

This is because the priests who confirm that Jesus is pure at the time of fasting also require people to first show themselves to the priests for confirmation. Confirmation is necessary. If there is sight, it confirms by seeing. If there is hearing, it confirms by listening. But when it comes to physical health matters, confirmation is needed, and you go to show yourself to the priests.

If the doctor and the patient say the medicine should not be stopped, then it should not be stopped; they will decide together.

It is very difficult. Let me tell you about what recently happened to me. The children of the church are very precious. In a very surprising way, she weakened at a time when we were praising God. We are happy because it’s said that sometimes you cannot avoid weakening for many reasons.

We gave thanks to God. It has been nine months full of struggle. They said Dr. Teneja said it’s a serious condition. And is it true that when someone loses movement on Sunday morning, no one notices? When you went, the heart had stopped beating and they said she needs to be resuscitated.

On Monday morning, she was waiting while being treated in the emergency room. We came in the evening. Since Sunday night, she has been slipping away, and she is very tired. She asked me again, “Why? We prayed and fasted, why now?”

I couldn’t get angry; I told her, “God knows your feelings better than I do.” She said, “My father is not here to help me,” and she kept saying this. I had no answer except to pray for her. We don’t have answers for everything.

Another woman was born the same way and later found out she has breast cancer. She was operated on. She is the one who told me a hundred things, she is tired and weakened. She is her first child.

I pray for her and encourage her to continue with her treatment, but what can I do? I have no answers for some things.

We are but vessels in God’s hand. He can do what He wills. All we can ask for is mercy. We know people who have survived cancer. We know those whom God has completely healed.

God’s grace is abundant. I don’t know how He strengthens these people. I don’t always have an answer. Life itself is a gift. You can’t say why or how He allows certain things to happen. Life returns to the Giver, and trying to find answers for these kinds of things can be exhausting. The reason is that unanswered questions remain inside us.

When people seek answers for these questions, they can feel lost or confused because there is no clear answer, and this causes frustration. I don’t know why this has come to pass. I do not believe God would punish parents by harming their eight-month-old child. I can’t say that. The Bible clearly says: “Fathers, eat the fruit of the vine,” which means to enjoy what God provides. So children’s teeth can sometimes be affected — this is a natural, unavoidable process.

I tell myself God will answer each person according to His plan and work. If God wishes to discipline, I cannot tell Him why or how He should punish His child. Nor can I provide an explanation. But He knows. God is righteous and fully aware of all that happens. I don’t know why He protected that child the way He did. But everything happens for a purpose.

I do not believe God does anything without meaning or control. Nothing is outside His authority. There is such a thing as evil’s temporary influence, but how long He allows it, I don’t know.

I do know we live in a world of trials. It is a fallen world full of sin and brokenness. I know the first sin caused great destruction. But God has made an eternal promise.

He is pure and righteous, and He knows the way forward. After that, there is no more pain, no more suffering — that’s what He says.

God wipes away every tear from our eyes.

Others say that medicine is what cures. They say healing comes from medicine. These people are criminals to me. When someone is sick, the person who can heal them causes them to die by preventing them from using medicine or even having a little of it. These people will shock you—when their own children get sick, they take what is called modern medicine first. When it comes to you, they say it’s your choice. Some even have business dealings with medicine. Some say, “No, it’s not allowed.” These people are above us and even the church in influence; you cannot say anything to them.
