# Supplementary material for: Negotiating illness through faith: Religious narratives of cancer etiology among patients and caregivers in Addis Ababa, Ethiopia
Source: PLoS One. 2026 Jun 5;21(6):e0349510. doi: 10.1371/journal.pone.0349510 (PMC13240894; doi:10.1371/journal.pone.0349510)
Supplement: Attachment — Submitted filename: Response to Reviewers.docx [file pone.0349510.s003.docx]

**Response to Reviewers**

We sincerely thank the Editor and the Reviewers for their careful reading of our manuscript and for their insightful and constructive comments. We appreciate the opportunity to revise and improve our work. We have carefully considered all suggestions and have revised the manuscript accordingly. Below, we provide a detailed, point-by-point response to each comment. Although we have revised or reworked the entire sections of the manuscript, all the major changes are highlighted in **Yellow.**

**Reviewer #1:** The manuscript addresses an underexplored dimension of cancer care in Ethiopia—religious interpretations and meaning-making among patients and caregivers. The topic is important and culturally relevant. However, the paper requires major revisions before it can meet the scientific and methodological. Key concerns relate to conceptual clarity, methodological rigor, analytic depth, and internal consistency.
Q1: Conceptual Framework: Not Well Defined
Although the study uses phenomenology, there is no clear theoretical or analytic framework guiding how “religious meaning-making” is conceptualized.
Q2. Why this is a problem: The manuscript repeatedly uses terms such as punishment, test of faith, sovereign plan, supernatural, etc., but does not explain how these constructs are defined or distinguished in existing scholarship.
Q3.The analysis risks becoming descriptive rather than theoretically grounded.

**Response:** We thank the reviewer for this important observation. We agree that the initial version of the manuscript did not sufficiently articulate the conceptual and theoretical foundations guiding our analysis of religious meaning-making, especially in the context of illness causation and suffering.

In response, we have substantially revised the Introduction and Discussion sections to clarify the analytic framework underpinning the study. Specifically, we now explicitly situate our analysis within:

1. Kleinman’s explanatory model framework, to conceptualize religious interpretations as culturally shaped accounts of illness causation and suffering;
2. Social constructionist perspectives, to explain how shared religious meanings are produced and sustained within communities;
3. Medical anthropological concepts of personalistic and naturalistic etiologies, to distinguish spiritual and biomedical explanations of illness; and
4. Spiritual coping frameworks, to interpret how beliefs such as “test of faith” or “divine plan” function in emotional regulation and resilience.

We have also added a dedicated subsection in the Methods/Conceptual Framework section explaining how key constructs (e.g., punishment, test of faith, divine plan, supernatural causation) are defined and analytically distinguished based on existing scholarship. These conceptual distinctions now guide our data coding and interpretation process.

Furthermore, the Discussion section has been revised to explicitly link empirical findings to these theoretical perspectives, strengthening the analytical depth and reducing purely descriptive interpretation as rightly indicated by the reviewer.

**Required Revision**
**Reviewer #1 comment:** Provide a conceptual model linking phenomenology, meaning-making, and religious explanatory models.
Clarify whether the study draws from: Kleinman’s explanatory models, Berger & Luckmann’s social construction, Personalistic/naturalistic etiologies, Spiritual coping frameworks

**Response 1:** We thank the reviewer for this valuable comment. We have revised the Introduction to clarify the theoretical foundations of the study and to present an integrated conceptual framework linking phenomenology, meaning-making, and religious explanatory models. Specifically, we now situate the study within Kleinman’s explanatory model framework, Berger and Luckmann’s social constructionist perspective, Foster’s *personalistic* etiologies, and spiritual coping theory (pp. 7, Para. 1). This addition clarifies how these perspectives guide our analysis.
**Reviewer #1 comment:** Q4. Methodological transparency is insufficient.
Several methodological elements require clarification or strengthening.
1. Sampling strategy is not sufficiently justified.
Why were only 6 cancer patients included but 33 caregivers?
Phenomenology typically emphasizes deep exploration with fewer participants, not imbalanced large samples.
2. No explanation of saturation.
How was thematic saturation reached?
3. Translation procedure insufficiently described.
Back-translation?
Were multiple coders involved?
4. Data analysis lacks rigor.
No clearly mention of software, coding reliability, reflexivity, or audit trails.

**Response:** We thank the reviewer for highlighting the need for greater methodological transparency.

1. We have substantially revised the Methods section to clarify the sampling strategy and justify the sample composition (pp. 10, Para. 1).

“Although the initial aim was to recruit a comparable number of patients and caregivers, the study included fewer patients due to illness severity, as most presented at advanced stages, treatment schedules, and fatigue during treatment administration, particularly during and after chemotherapy. In contrast, caregivers were more accessible, interested, and actively involved in care decisions and spiritual practices. Given the study's focus on relational meaning-making within families, caregivers were retained as key participants.”

2. We have also added a detailed description of how thematic saturation was assessed (pp. 12, Para, 2),

“Participants’ recruitments continued until data saturation was reached, a point at which additional interviews yielded limited new insights and the dataset supported a coherent interpretive account [39]. Data saturation was assessed through ongoing review of interview transcripts and emerging codes. Saturation was reached after approximately 41 interviews with patients, caregivers, and religious figures, at which point further data collection was discontinued.”

3. Strengthened the translation and verification procedures (p. 11-13), and expanded the data analysis section to include information on coding procedures, use of software, reflexivity, and audit trails (pp. 12-13). These revisions enhance the rigor and transparency of the study.

Q 5: **Reviewer Comment:**
Over-reliance on Bible/Qur’an quotations. The manuscript includes lengthy scripture citations, which risk shifting the paper from academic to theological writing. PLOS ONE requires scientific—not doctrinal—analysis. Quotations crowd the Results section and reduce analytic clarity. Religious texts should be interpreted sociologically, not reproduced at length.

**Response:** We thank the reviewer for this important and constructive comment. We agree that the initial version of the manuscript relied too heavily on lengthy scriptural quotations, which may have shifted the focus from sociological analysis to theological exposition.

In response, we have carefully revised the Results and Discussion sections to substantially reduce the length and frequency of scriptural citations, focusing only on how participants used and interpreted the texts in meaning-making processes of their situations, rather than to present scriptural tests. Extended quotations have been removed or condensed and replaced with concise paraphrases that highlight their sociological significance rather than their doctrinal content.

We have also strengthened our interpretive commentary by explicitly linking participants’ use of scripture to relevant theoretical frameworks, including explanatory models, social constructionist perspectives, and spiritual coping theory.

Q6: **Reviewer Comment:** Ethical statement needs correction. The manuscript states that no minors participated, yet Table 1 includes children with cancer and caregivers of children. This is a serious inconsistency. Clearly explain assent and parental consent procedures for minors.

**Response:** We thank the reviewer for identifying this important inconsistency. We clarify that no minors were directly recruited or interviewed in this study. All interview participants were adults (≥18 years), including adult patients, adult family caregivers, and religious leaders.

The reference to “childhood cancer” in Table 1 reflects the age category of the patient being cared for, as reported by adult caregivers, rather than the age of interviewed participants. In other words, caregivers of pediatric patients were interviewed, but children themselves were not enrolled in the study.

We have revised the Ethical Considerations and Methods sections to clearly state that only adults participated in interviews. We have also revised Table 1 and its caption to clarify that age categories refer to the patients’ age at diagnosis, not the age of respondents.

Since no minors were enrolled, assent procedures were not required. Written informed consent was obtained from all adult participants.

Q7. **Reviewer Comment:**
Table 1 errors. The table header does not specify whether N refers to patients only, caregivers only, or total participants. Percentages cannot be interpreted if the denominator is unclear. Table 1 needs amendment.

**Response:** We thank the reviewer for this important observation. We agree that the original version of Table 1 did not clearly specify the reference population for N and percentages, which may have limited interpretability.

In response, we have revised Table 1 to explicitly indicate the number of participants in each category (patients, caregivers, and religious leaders) and to clarify the denominators used for percentage calculations. The table header and footnotes now specify whether percentages are calculated based on the total sample or relevant subgroups.

Specifically, we have included:

- The total number of participants (N = 41),
- The number of patients (n = 6), caregivers (n = 33), and religious leaders (n = 2), and
- Separate percentage calculations for each subgroup where applicable,

Q8: **Reviewer Comment:**

How did the authors ensure that data saturation was truly achieved, given the limited number of patient interviews and the heterogeneity of religious, gender, and contextual backgrounds?”

**Response:** We thank the reviewer for raising this important point regarding saturation. As has been stated earlier, we have revised the Methods section to clarify how saturation was systematically assessed. Data collection and analysis were conducted concurrently, and emerging themes were continuously compared across interviews and participant groups. Although the number of patient interviews was limited, in-depth idiographic analysis revealed strong convergence in core meaning-making patterns. Recruitment continued until successive interviews, including those from diverse religious and gender backgrounds, yielded no substantively new insights. I also assessed saturation across patients, caregivers, and religious leaders, which indicated thematic stability. These procedures are now described in detail on pp. 12.

**Reviewer # 2: Comment:** The inclusion of religious leaders muddies the paper and takes away from the focus. I suggest removing religious leaders and discussions about religious texts and focusing on participants’ perspectives.

**Response:**
We thank the reviewer for this thoughtful and constructive suggestion. We agree that the primary focus of this study is on the lived experiences and meaning-making processes of patients and family caregivers.

However, we included religious leaders as key informants to provide contextual insight into the religious discourses and interpretive frameworks that shape participants’ understandings of illness. Their perspectives helped to situate patients’ and caregivers’ narratives within broader community and institutional religious contexts.

In response to the reviewer’s concern, we have revised the manuscript to strengthen the centrality of patients’ and caregivers’ voices. Specifically, we have reduced the prominence of religious leaders’ accounts, limited their quotations, and ensured that they are used only to contextualize—rather than dominate—the main findings. We have also removed extended discussions of religious texts and shifted the analytical emphasis to how participants interpret and mobilize these texts in their own narratives.

These revisions sharpen the analytical focus of the manuscript while retaining the limited inclusion of religious leaders as contextual informants, thereby enhancing the depth and interpretive rigor of the study.

**Comment:** Page five first paragraph: “emphasizing on” Do you mean emphasis on?

**Response:** Thank you for noting this. We have corrected “emphasizing on” to “emphasis on” in the revised manuscript.

**Reviewer’s Comment:** I may have missed something, but I think the last paragraph before the methods section is the first time qualitatively data with religious figures is mentioned. Most (if not all) earlier discussions focus on patients and caregivers.

**Response:** We thank the reviewer for this important observation. We would like to clarify that religious figures were not the primary focus of this study. The central analytical emphasis remains on patients’ and caregivers’ experiences and meaning-making processes. Religious figures were included to get a perspective on how religious teachings, practices, and traditions shape and inform patients’ and caregivers’ interpretations of illness and care-seeking. In the revised manuscript, we have clarified this rationale in the Introduction and Methods sections to better reflect their supporting role in the analysis. Besides, we have reduced the prominence of religious leaders’ accounts, limited their quotations, and ensured that they are used only to contextualize rather than dominate the main findings. We have also removed extended discussions of religious texts and shifted the analytical emphasis to how participants interpret and mobilize these texts in their own narratives.

**Reviewer’s Comment:** On the bottom of page 5, there might be a word or two missing. As it reads now, the sentence is a fragment. The same thing with the first sentence of the first full paragraph on page 6.

**Responses:** Thank you for bringing this to our attention. We have carefully reviewed the indicated sections and revised both sentences to correct the fragments and ensure grammatical completeness and clarity.

**Reviewer’s comment:** The last sentence of the first full paragraph on page 6 only mentions caregivers. The next section talks about the participants, so I’m not sure you need this last sentence where it’s at. If you keep it, add all of the types of people interviewed.

**Response:** Thank you for this helpful suggestion. We agree that the placement and wording of this sentence could be misleading. In response, we have revised this sentence to include all participants interviewed in the study and clarified its connection to the following section. Alternatively, where appropriate, we have removed the sentence to improve coherence and flow.

**Reviewer’s comment:** I’m not sure perspectives from religious leaders fits within your study. You are focusing on people’s everyday lived experiences, not leaders’ perspectives on something.

**Response:** We thank the reviewer for raising this important point. We agree that the primary focus of this study is on patients’ and caregivers’ everyday lived experiences. The perspectives of religious leaders were not included as a separate analytical focus, but rather to provide contextual insight into the religious teachings, practices, and traditions that shape participants’ interpretations of illness and care-seeking.

Their inclusion was intended to enhance understanding of the broader religious and cultural environment within which patients and caregivers construct meaning, rather than to shift the analytical emphasis away from lived experience. In the revised manuscript, we have clarified this rationale and ensured that the Results section continues to prioritize patients’ and caregivers’ narratives.

**Reviewer’s comment:** At the bottom of page 6: Where did the nurse practitioner do this recruiting? Talking individually to her patients? At a class or seminar? A signup sheet?

**Response:** Thank you for requesting clarification on the recruitment process. Initial contact with potential participants was facilitated individually by an oncology nurse during routine clinical encounters. Patients and caregivers were approached in person, informed about the study, and invited to participate. Recruitment did not occur through group sessions, classes, seminars, or public sign-up sheets. We have clarified this procedure in the revised Methods section.

**Reviewer’s comment:** Page 7: data were, not data was

**Response:** Thank you for noting this. We have corrected “data was” to “data were” in the revised manuscript.

**Reviewer’s comment:** I’m a bit confused by Table 8. 18 children are caregivers? There’s only 6 actual patients in your study? I’m not sure what the mother and father in parentheses for individuals diagnosed with cancer means.

**Responses:** Thank you for highlighting this issue. We apologize for the lack of clarity in Table 1. In response, we have revised Table 1 to explicitly indicate the number of participants in each category (patients, caregivers, and religious leaders) and to clarify the denominators used for percentage calculations. The category “children” refers to adult children who were providing care to a parent diagnosed with cancer, rather than child caregivers.

The number of patients has also been clarified to avoid misinterpretation. Additionally, we have revised the use of “mother” and “father” in parentheses to indicate their relationship to the caregiver participant, rather than suggesting that they were separate study participants. These revisions have been made to improve accuracy and readability.

**Reviewer’s comment:** It looks like your study mainly focuses on people who were very recently diagnosed with cancer. I would imagine that caregivers’ perspectives might change as time goes on and even patients’ perspectives. You may want to set this up in your intro and lit review that you are focusing on people very recently diagnosed with cancer. Years since initial diagnosis

**Response:** We thank the reviewer for this important observation. We would like to clarify that, although some participants were recently diagnosed, the majority of our sample had been living with a cancer diagnosis for one year or longer. Specifically, 17 participants had been diagnosed for more than one year at the time of interview.

| **Years since initial diagnosis**  >3months  4-6months  7months-1years  >1year | 8  10  4  17 |
| --- | --- |

In response, we have revised the manuscript to more clearly present the distribution of time since diagnosis and to emphasize that the study captures perspectives from both early and longer-term stages of illness experience. Besides, we have further addressed this issue in the Limitations section by noting that perspectives may change over time and that future research could explore longer-term experiences.

**Reviewer’s comment:** How does confidentiality and anonymity in how patients are treated affect “bias” in recruitment? Unless customs are different, you don’t need to address “bias” in a phenomenological study.

**Response:** We thank the reviewer for this important observation. We agree that, in phenomenological research, the primary concern is not statistical representativeness or sampling “bias,” but rather the depth and richness of participants’ lived experiences.

Our discussion of confidentiality and anonymity was intended to demonstrate ethical rigor and to explain how trust was established with participants, rather than to suggest the elimination of recruitment bias. In response, we have revised the manuscript to clarify this point and to avoid unnecessary emphasis on “bias” in the context of a phenomenological design.

**Reviewer’s comment:** The last sentence before the Results section is odd.

**Response:** Thank you for noting this. We agree and have revised this sentence for clarity and coherence and to ensure a smooth transition into the Results section.

**Reviewer’s comment:** Did your interview guide include questions specifically about religion or did you not mention anything about religion and it emerged organically from the interviews?

**Response:** Thank you for this important question. The interview guide included open-ended questions about participants’ illness experiences, meanings, and care-seeking practices. While religion was not always introduced as a standalone topic at the beginning of interviews, participants were encouraged to reflect on spiritual or religious beliefs when these emerged naturally in their narratives. In some interviews, follow-up prompts were used to explore religious interpretations in greater depth when participants introduced these themes.

In the revised manuscript, we have clarified this approach in the Methods section to better reflect how religious narratives were elicited (pp. 12, Para. 1, line 8-11).

**Reviewer’s comment:** The second part of the first quote in the results section doesn’t sound like an interview quote. It sounds like your analysis. Again, unless customs are different, separate your analysis from the quote.

**Response:** We thank the reviewer for this important observation. In response, we have revised and replaced it with the participant’s original verbatim words

**Reviewer’s comment:** I’m not following the organization of the Results section. Why is there a section 1 that is separate from the subthemes you are discussing? The focus should be on the subthemes. It makes sense to generally talk about the importance of religion before you mention the subthemes, but you don’t need a “section” to do that.

**Response:** We thank the reviewer for this helpful comment. We agree that the Results section should focus primarily on the identified subthemes. In response, we have removed the standalone “Section 1” and integrated the general discussion of the importance of religion into the introductory text of the Results section. This material now serves as contextual framing for the subthemes rather than as a separate section.

The Results section has been reorganized accordingly to improve clarity, coherence, and alignment with qualitative reporting standards.

**Reviewer’s comment:** On page 17: You described the story of the blind man earlier, so you don’t need to provide an actual quote from the Bible. Your focus is on how people interpret and use religious teachings, not what the “actual” teachings say.

**Responses:** We thank the reviewer for this helpful comment. We agree that the focus of the study is on participants’ interpretations and uses of religious teachings rather than on the original biblical text itself. In response, we have removed the direct quotation from the Bible and retained only participants’ interpretations and references to the story in the Results section.

**Reviewer’s comment:** Is the passage in the book of Job mentioned by any of the participants? If not, you don’t need to focus on it.

**Response:** We thank the reviewer for this important observation. The passage from the Book of Job was not directly mentioned by participants but was included as contextual background. We agree that this may detract from the primary focus on participants’ narratives. In response, we have removed (or substantially reduced) this discussion and refocused the section on how participants themselves interpreted and referenced religious teachings.

**Reviewer’s comment:** At the top of page 20: Again, did any of the participants invoke that passage as a way to understand their illness? That is the purpose of your study. Top of page 21: same thing. You are talking about scripture, but not any of your participants who invoke that scripture. Page 22-23: same thing. I don’t think the religious leaders add to your study. They take away from its focus. Page 23-24: same thing. Pages 24-27: Same thing. Focus the study around what your participants say and what scripture they invoke. I “think” that is the purpose of your study.

**Response:** We thank the reviewer for this thoughtful and detailed comment. We agree that the primary purpose of this study is to examine how participants themselves interpret, invoke, and use religious teachings in making sense of illness.

In response, we have revised pages 20–27 to substantially reduce discussions of scripture and religious teachings that were not directly referenced by participants. We have refocused the analysis on participants’ own narratives and on the specific religious passages and concepts they explicitly invoked.

We have also minimized the use of religious leaders’ perspectives to ensure that the Results section remains centered on patients’ and caregivers’ lived experiences and meaning-making processes. These revisions strengthen the analytical coherence of the manuscript and better align it with the study’s phenomenological focus.

**Reviewer’s comment:** At the top of page 31: You talk about different meanings and interpretations within the same family, but I don’t recall your data discussing or demonstrating this idea.

**Responses:** We thank the reviewer for this observation. We would like to clarify that the intended point was not about differences between family members, but rather that individual participants—whether patients or caregivers—sometimes entertained multiple or even conflicting narratives about the causes of illness and the role of religion in care-seeking.

In response, we have revised the text on page 31 to make this distinction explicit and ensure that it is clearly grounded in participants’ accounts, avoiding any unsupported claims about intra-family variation.

**Reviewer’s comment:** At the top of page 32: You are talking about what different religions teach rather than reiterating patterns of how your participants make meaning from religion.

**Response:** We thank the reviewer for this important observation. We agree that the focus of the Results section should remain on participants’ meaning-making rather than on general religious teachings. In response, we have revised the section at the top of page 32 to remove descriptive content about what different religions teach and instead emphasize patterns in how participants interpret and use religious beliefs to understand illness and guide care-seeking.

**Reviewer’s comment:** On page 32: You have a very interesting discussion of how Muslims Vs. Christians use different ideas to make meaning out of cancer. I don’t recall this point being made in your findings or the differences highlighted/organized this way in your findings. This is a very interesting point, so you may want to consider emphasizing it in your findings.

**Response:** We thank the reviewer for highlighting this insightful point. We agree that the discussion on differences between how Muslim and Christian participants interpret and use religious ideas is interesting. To strengthen the manuscript, we have revised the Results section to explicitly present these patterns, organizing the findings to highlight how participants from different religious backgrounds make meaning of cancer. This ensures that the discussion in the later sections is clearly grounded in the data presented in the finding. See it from pp. 17-19.

**Reviewer’s comment:** On page 33: I don’t think you need the limitation of diverse religious leaders. Rather, I think you can talk about research with religious leaders as a suggestion for future research.

**Responses:** We thank the reviewer for this helpful suggestion. We agree that the inclusion of diverse religious leaders is not a limitation of the current study, since the focus remains on patients’ and caregivers’ lived experiences. In response, we have removed this point from the limitations section, and instead suggested that future research could explore the perspectives of religious leaders to further understand how religious teachings shape illness meaning-making.

Reviewer comment: You may want to look at Rachel Kraus’s work (in Sociology of Religion and Journal for the Scientific Study of Religion). She researches specifically how women with metastatic breast cancer use religion to make meaning out of their experiences.

**Response:** We thank the reviewer for this helpful suggestion. We have reviewed the work of Rachel Kraus, particularly her studies published in Sociology of Religion and Journal for the Scientific Study of Religion. Her research on how women with metastatic breast cancer use religion to make meaning of their experiences aligns closely with our findings on the role of spiritual and religious frameworks in shaping interpretations of illness. We have now incorporated discussion of her work in the manuscript (see page 35) to contextualize our findings within the broader literature on religion, meaning-making, and coping in serious illness.
