# Supplementary material for: Negotiating illness through faith: Religious narratives of cancer etiology among patients and caregivers in Addis Ababa, Ethiopia
Source: PLoS One. 2026 Jun 5;21(6):e0349510. doi: 10.1371/journal.pone.0349510 (PMC13240894; doi:10.1371/journal.pone.0349510)
Supplement: Attachment — Submitted filename: Response_to_Reviewers_auresp_2.docx [file pone.0349510.s004.docx]

**Response to Reviewers**

**We sincerely thank the Editor and the Reviewers for their careful reading of our manuscript and for their insightful and constructive comments. We appreciate the opportunity to revise and improve our work. We have carefully considered all suggestions and have revised the manuscript accordingly. Below, we provide a detailed, point-by-point response to each comment. Although we have revised or reworked the entire sections of the manuscript, all the major changes are highlighted in Yellow.**

**Comments from Reviewer #2:** Data collection: the fact that you did not have pre-existing questions regarding religion is a big deal. It is telling that your interviewees brought religion up without prompt. You may want to consider emphasizing this point more.

**Response:**
Thank you for this insightful observation. We agree that the spontaneous emergence of religious narratives during interviews is an important finding in itself. In response, we have revised the manuscript to clarify that religion was not included as a pre-defined focus in the interview guide. Instead, participants independently raised religious explanations and interpretations when discussing their illness experiences. We now emphasize this point more explicitly in the data collection section, as it highlights the salience and centrality of religion in participants’ meaning-making processes. See it from the revised manuscript pp. 12, highlighted in yellow.

**Reviewer Comment 1**
*On page 15: I’m not sure about your discussion of transferability. What is written is very vague and doesn’t get at how/whether your study would be applicable in somewhat similar and different contexts. You may want to consider a transferability discussion in the suggestions for future research.*

**Response 1**
Thank you for this insightful comment. We have substantially revised the manuscript to strengthen the discussion of transferability. Specifically, we have clarified how the findings are contextually grounded and articulated the conditions under which they may be relevant to other settings, particularly those with similar sociocultural and healthcare characteristics.

In addition, we have explicitly addressed transferability in two key sections. First, we have incorporated a more focused discussion in the **Limitations** section, emphasizing the context-specific nature of the findings and the potential constraints in applying them to more secular or structurally different healthcare environments. Second, we have expanded the **Implications/Future Research** section to outline how comparative and cross-contextual studies could further assess and extend the transferability of these findings.

We have also refined the **trustworthiness** section by providing a more concrete account of how transferability was supported through rich, contextualized descriptions of the study setting and participants, enabling readers to assess relevance to other contexts.

We believe these revisions address the reviewer’s concern and improve the clarity and rigor of the manuscript. *See the changes from pp. 15 section: Trustworthiness of the study; pp. 37, section Implication of the study; pp.38, section Limitations of the study, all highlighted in Yellow.*

**Reviewer Comment 2**
*Page 17: I don’t think you need the box. The “theme” isn’t really a finding. It’s more of a Question. I do think a summary paragraph at the beginning of a findings section is helpful, so if you remove the box, you can mention the subthemes in a paragraph.*

**Response 2**
Thank you for this helpful suggestion. We agree that the table did not effectively present the analytical contribution of this section. In response, we have removed the table and revised the Results section to begin with a concise summary paragraph that synthesizes the key findings.

Specifically, we now introduce the subthemes as analytically derived interpretations—namely, cancer as divine punishment, a test of faith, a manifestation of divine or sovereign will, and as an attack by evil spirits or Satan—rather than presenting them as items within a table. This restructuring allows for a more coherent and interpretive presentation of the findings and improves the overall narrative flow of the section.

We believe this revision strengthens the clarity and analytical depth of the manuscript and better aligns with qualitative reporting standards. *See the changes from the results section first paragraph, pp.15, highlighted in yellow.*

**Reviewer Comment 3**
*On page 17: it doesn’t make sense that the quote you provide was provided by multiple participants. It’s quite ok to talk about the general pattern and provide a quote that illustrates the pattern.*

**Response 3:**
Thank you for this important comment. We agree that the previous version did not clearly distinguish between the general analytic pattern and the illustrative role of individual quotations. In response, we have revised the section to first present the shared pattern identified across participants in our analytic voice, followed by a single participant quotation used strictly as an illustration of this pattern.

We have also removed phrasing that implied the quotation represented multiple participants and revised the surrounding text to improve clarity and consistency in the presentation of findings. These changes enhance the transparency and rigor of the qualitative analysis and align the manuscript with established conventions for reporting qualitative data. See the changes from pp. 17, highlighted in a yellow.

**Reviewer Comment 4:**
Page 18: who is “her” in this sentence? The referent is unclear.

**Response 4:**
Thank you for pointing out this ambiguity. We agree that the original wording was unclear regarding the referent of “her.” We have revised the paragraph to clearly distinguish between the caregiver (the participant) and the patient (her mother), ensuring consistent and precise attribution throughout the narrative. These revisions improve clarity and avoid any potential confusion in interpreting the quotation and its accompanying analysis. See the changes from pp. 19, highlighted in a yellow.

**Reviewer Comment 5:**

*Page 19 second paragraph: what is “it”?*

**Response:**
Thank you for this comment. We agree that the previous use of the pronoun “it” was unclear. We have revised the sentence for clarity by replacing the ambiguous reference with a precise subject. The revised version now reads: “These interpretations linked illness and suffering to perceived moral transgressions, reinforcing understandings of cancer as spiritually consequential.” This revision improves grammatical clarity and strengthens the conceptual precision of the paragraph.

**Reviewer Comment 6:**
*Page 19 last paragraph: What you have here is an EXCELLENT justification for why you include some commentary from religious leaders. It may help to emphasize this connection early on in the paper.*

**Response 6:**
Thank you for this valuable suggestion. We agree that the role of religious leaders and broader religious institutions in shaping illness meanings needed to be made more explicit at an earlier stage. In response, we have revised the opening of the “Religious Narratives of Illness” section to clearly indicate that participants’ interpretations of cancer are not only individually constructed but also shaped and reinforced through broader social and institutional religious influences.

Specifically, we have added a framing paragraph that highlights the role of religious leaders, communal worship, and pastoral counseling in reinforcing moral and spiritual interpretations of illness. This revision strengthens the conceptual coherence of the section and clarifies the importance of social and institutional contexts in shaping participants’ meaning-making processes.

**Reviewer Comment 7**

*Page 20: pastor pastor’s*

**Response 7**
Thank you for this comment and we have corrected the phrasing for grammatical accuracy and clarity by removing the repetition and ensuring correct possessive usage. The sentence has been revised to clearly indicate that the explanation is attributed to a single pastor, thereby improving readability and precision in the presentation of the findings.

**Reviewer Comment 8**

*Page 25: I’m not understanding the “origin of cancer and its diagnosis”*

**Response: 8**
Thank you for this comment. We agree that the previous wording was unclear. We have revised the sentence for clarity and conceptual precision. The phrase “origin of cancer and its diagnosis” has been removed and replaced with “cancer” to better align with the focus on illness narratives and meaning-making. The revised sentence now reads: “Another prominent illness narrative interpreted cancer as part of a divine sovereign plan.” We believe this improves clarity and strengthens the analytical framing of the findings.
